# Racial Differences in the Pathway to Diagnosis of Alzheimer's Disease and Related Dementias

**DOI:** 10.1093/geroni/igab046.1082

**Published:** 2021-12-17

**Authors:** Hanzhang Xu, James Burke, Matthew Dupre, Se Hee Min, Ruth Anderson, David Page, Truls Ostbye

**Affiliations:** 1 Duke University School of Medicine, Duke University, North Carolina, United States; 2 Duke University School of Medicine, Durham, North Carolina, United States; 3 Duke University School of Nursing, Durham, North Carolina, United States; 4 University of North Carolina at Chapel Hill, UNC Chapel Hill, North Carolina, United States; 5 Duke University, Durham, North Carolina, United States

## Abstract

This study examined differences in the pathway to diagnosis of Alzheimer's disease and related dementias (ADRD) between Black and White older adults. Using electronic health records from a large health system, we included 2,085 non-Hispanic Black and 6,269 non-Hispanic White older adults with a final/primary diagnosis of ADRD between 2014 and 2020. Black older adults were more likely to receive the ADRD diagnosis from a primary care provider (35.4% vs. 29.8%), during a hospital admission (19.5% vs. 13.6%), or during an emergency department visit (4.2% vs. 2.0%); but were less likely to be diagnosed by an ADRD specialist (31.6% vs. 45.2%). Black older adults had nearly twice as many clinical encounters in the two years prior to the ADRD diagnosis than their White counterparts (43 vs. 26). Despite having more clinical encounters, Black older adults were more likely to be at a later stage when diagnosed than White older adults.

